# The Association of Previous Day Carbohydrate Consumption With Fasted, Exhaled Carbon Dioxide in Lumen Users: Retrospective Real-World Study

**DOI:** 10.2196/64604

**Published:** 2025-09-02

**Authors:** Shlomo Yeshurun, Tomer Cramer, Daniel Souroujon, Merav Mor

**Affiliations:** 1 Metaflow Ltd Tel Aviv Israel; 2 School of Public Health Tel Aviv University Tel Aviv Israel

**Keywords:** Lumen, fasting, carbohydrate, mHealth, BMI, metabolism

## Abstract

**Background:**

The increasing prevalence of obesity and related metabolic disorders has highlighted the need for innovative tools to monitor and manage metabolic health. The Lumen device offers a novel approach to assess the metabolic state through exhaled carbon dioxide (CO_2_) measurements, providing real-time feedback via a mobile app. This app-driven experience allows users to track their metabolic state and receive personalized nutrition and lifestyle recommendations, potentially supporting long-term metabolic health improvements.

**Objective:**

This study aimed to investigate the association between the previous day’s carbohydrate consumption with fasted, exhaled CO_2_ levels in female and male Lumen users while also examining the influence of fasting duration, BMI, and age.

**Methods:**

We conducted a retrospective, observational study using deidentified data from 48,058 Lumen users, comprising 707,372 fasted sessions. Separate linear mixed models were fitted for female (n=520,269 sessions) and male (n=187,103 sessions) users due to observed sex differences in metabolism. User ID was included as a random effect to account for repeated measures. The models analyzed the relationship between fasted %CO_2_ levels and reported carbohydrate intake, fasting duration, BMI, and age.

**Results:**

Higher reported carbohydrate intake from the previous day was significantly associated with increased morning %CO_2_ levels in both male and female users (β=.032; *P*<.001; Cohen *d*=0.0691 in women and β=.024; *P*<.001; Cohen *d*=0.0534 in men), while a longer fasting duration was linked to decreased %CO_2_ levels in both sexes (β=−.017; *P*<.001 for both and Cohen *d*=−0.0374 in women and Cohen *d*=−0.0382 in men). A higher BMI was associated with elevated %CO_2_ levels in both sexes (β=.018; *P*<.001; Cohen *d*=0.0390 in women and β=.017; *P*<.001; Cohen *d*=0.0384 in men). Age had a statistically significant but modest effect in women (β=.008; *P*<.001; Cohen *d*=0.0180), whereas the effect size was minimal and did not meet the stricter significance threshold in men (β=.001; *P*=.02; Cohen *d*=0.0016). Cohen *d* values indicated that reported carbohydrate intake had the strongest effect size, while fasting duration and BMI had relatively smaller effects in both models.

**Conclusions:**

The Lumen device is able to detect changes in fasted %CO_2_ levels based on the previous day’s reported carbohydrate intake and fasting duration with sex-specific metabolic responses. These findings highlight the potential of Lumen as a personalized metabolic health monitoring tool, providing insights into the influence of dietary intake and fasting on metabolic state. Future research should investigate the hormonal and physiological mechanisms contributing to the observed sex differences and assess the long-term impact of app-guided metabolic feedback on user behaviors and metabolic health outcomes.

## Introduction

### Background

There has been a dramatic increase in the prevalence of obesity in recent years, posing significant health challenges globally [1,2]. Obesity is closely linked to an elevated risk of chronic diseases, including type 2 diabetes and cardiovascular diseases, which are leading causes of mortality worldwide [3,4]. Despite efforts to promote lifestyle changes and reduce caloric intake, sustained weight management remains challenging for many individuals, highlighting the need for innovative and effective interventions [5,6].

It has been proposed that modifying the macronutrient composition of the diet could induce weight loss [7,8]. For instance, diets low in carbohydrates or high in protein have shown promise in promoting satiety and supporting weight loss [9-11]. By modulating macronutrient intake, individuals may experience improved metabolic flexibility, which is the body’s ability to efficiently switch between burning carbohydrates and fats for energy, enabling better regulation of blood glucose levels and lipid metabolism [12,13]. Metabolic flexibility can be assessed by measuring the respiratory exchange ratio (RER) from metabolic cart measurements, where a lower RER during fasted states indicates greater reliance on fat oxidation over carbohydrate oxidation [13]. Short-term fasting has been shown to decrease RER values, reflecting the shift toward lipid metabolism and improved metabolic flexibility [14,15]. Metabolic flexibility is also linked to obesity, as individuals with a higher BMI are less flexible and more likely to experience metabolic disorders [16-19]. Therefore, enhancing metabolic flexibility through dietary adjustments may offer a viable strategy for mitigating the adverse effects of obesity.

Time-restricted eating is another recently popular diet, in which meals are limited to a specific period during the day. Several studies have shown that it is beneficial in weight loss, as well as other health benefits such as improved insulin sensitivity, reduced inflammation, and higher metabolic flexibility [20-22]. In addition, it may help to lower the risk of chronic diseases such as heart disease and diabetes [23]. Time-restricted eating has also been shown to decrease RER values during the fasted state, indicating a shift toward greater fat oxidation [24]. This reduction in RER during fasting periods suggests an enhanced capacity to use fat as a fuel source, which may contribute to the beneficial metabolic effects of intermittent fasting regimens [25].

Mobile health technologies, such as the Lumen breath analyzer, present innovative opportunities for real-time monitoring of metabolic responses to dietary changes [26,27]. Moreover, we recently showed that increased carbohydrate consumption in meals was associated with increased postprandial %CO_2_ response as measured by Lumen [28]. Similarly, applying continuous glucose monitoring (CGM) system with a mobile app was associated with improved glycemic control [29]. Such technologies empower users to make informed decisions about their diet and lifestyle, thereby potentially enhancing long-term health outcomes [30-33].

### Objective

This study aimed to investigate whether fasted %CO_2_ measurements are associated with the previous day’s carbohydrate intake, fasting duration as logged by Lumen users, and users’ age, sex, and BMI.

## Methods

### Study Design

This is a retrospective observational study based on deidentified data collected from users of the Lumen device and app.

### Participants

Participants’ data were collected retrospectively between September 16, 2023, and March 14, 2024. All users in the analysis were aged ≥18 years. As only 150 users in the database were found to be underweight (BMI ≤18.5), they were removed from the analysis.

### Data Sources

**Figure 1:**
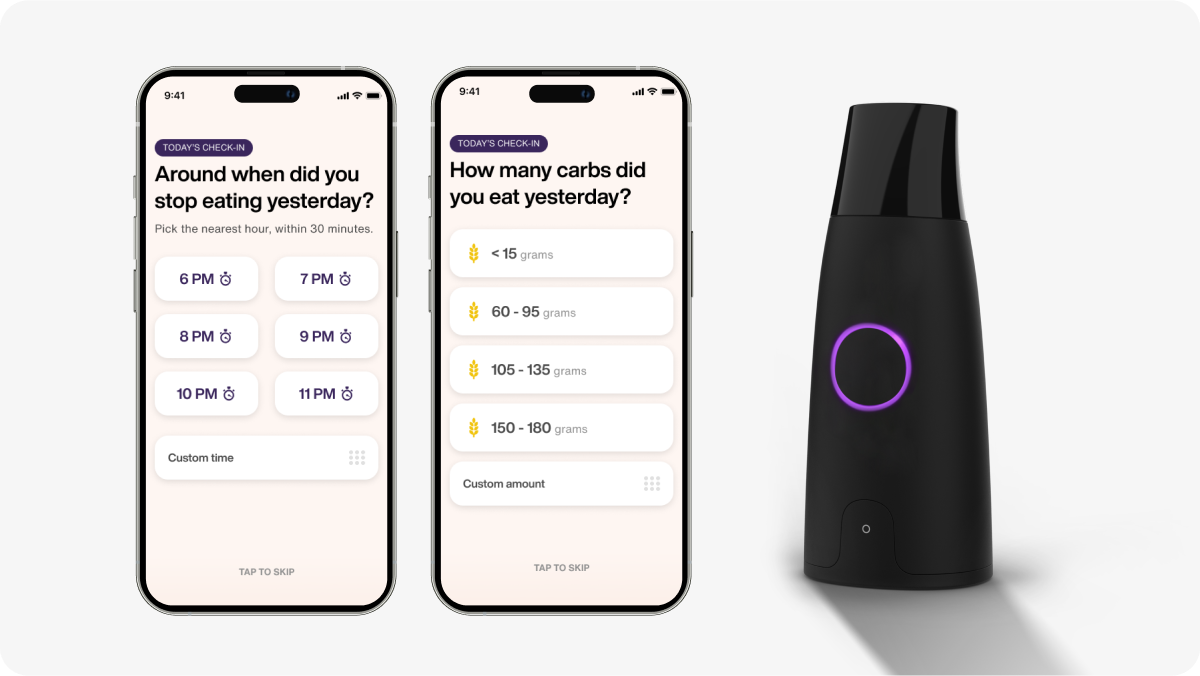
Lumen mobile app screenshots (A and B) detailing the user input asked for following a fasted %CO_2_ measurement with the Lumen device. (C) An illustration of the Lumen device.

During the onboarding process, users had to specify their sex (assigned at birth), date of birth, height, and weight. The Lumen app allows users to perform a variety of optional measurements with the Lumen device (MetaFlow Ltd), such as morning fasted, as well as pre- and postmeal measurements and a bedtime measurement before sleeping. The app guides the user through each phase of the breathing maneuver, and exhaled CO_2_ levels are obtained, as previously described [26]. The app provides nutritional recommendations based on the user’s personal preferences and their morning %CO_2_ measurements regarding how much macronutrients should be consumed during the day. In addition, with each morning measurement, the app asks users about the number of portions of carbohydrates they consumed the day before (1 portion=15 g) and when was the last time they consumed any food. An example of the morning questions in the app is shown in Figure 1. For this study, only fasted measurements where users reported both their carbohydrate intake and the time they stopped eating the previous day before were included. To avoid discrepancies, we removed the top and bottom 2.5% of data points for both the amount of carbohydrate portions consumed the previous day, and the duration of fasting hours, effectively eliminating outliers from both extremes. Moreover, in instances where more than half of a user’s measurements were removed for these reasons, that user’s entire dataset was consequently excluded from the analysis.

### Statistical Analysis

Data were analyzed using R Studio (version 2023.12.1; Posit PBC), and all statistical analyses were conducted with the R (version 4.3.3; R Core Team) programming language, using custom scripts and the *lme4* package [34].

Linear mixed models were fitted separately for women and men to explore how the previous day’s carbohydrate consumption, fasting duration, and personal characteristics (age and BMI) influence fasted %CO_2_ levels. Separating the sexes for analysis was based on well-documented metabolic differences and our previous work, which identified distinct %CO_2_ patterns in female Lumen users related to menstrual cycles and menopause [35]. Users’ unique IDs were included as a random effect to account for individual variability. Model fit was evaluated using Akaike Information Criterion, Bayesian Information Criterion, and *R*² values, with detailed results provided in Table S1 in Multimedia Appendix 1. Full random effects results and diagnostic checks are also presented in Table S1 and Figure S1 in Multimedia Appendix 1. Effect sizes were quantified using Cohen *d* to assess the relative strength of each predictor variable in explaining %CO_2_ variation.

Among all performed analysis, a 2-sided *P*≤.01 was considered statistically significant, following Maier and Lakens [36] and Wulff and Taylor [37] to mitigate inflated type I errors in large datasets and ensure robust findings.

### Ethical Considerations

This study was determined to be exempt from institutional review board (IRB) under category 2, as detailed in 45 CFR 46.104(d) and the standard operating procedure of the Biomedical Research Alliance of New York (BRANY), by the BRANY Social, Behavioral, and Educational Research IRB on May 9, 2023 (BRANY IRB File 23-119-1476). Exemption was granted because the study involved the secondary use of deidentified data that were originally collected for purposes other than this research, ensuring that participants could not be identified. All user data were fully anonymized to minimize the risk of violating participants’ privacy, and no identifiable information was accessible to the researchers. As such, informed consent was not required, and no compensation was given to the participants.

## Results

### Participants

Overall, 48,058 users have completed 707,372 fasted sessions reporting their carbohydrate intake from the previous day. These sessions were included in the final analysis, with a median of 8 (IQR 6-10) sessions per user.

### Descriptive Characteristics

Among the users, 71.24% (34,238/48,058) were women. Female participants were slightly older than their male counterparts, with a mean age of 49.9 (SD 10) years compared to 48.1 (SD 10.2) years, and had a slightly lower BMI, averaging 29.2 (SD 6) kg/m^2^ compared to 30.4 (SD 5.3) kg/m^2^. A summary of the participants’ characteristics is presented in Table 1.

Across 707,372 morning measurements, users reported consuming an average of 7.9 (SD 3) portions of carbohydrates the previous day. Women consumed an average of 7.5 (SD 2.7) portions, while men consumed 9.1 (SD 3.5) portions. The average fasting duration was 11.3 (SD 1.8) hours, with women fasting for 11.3 (SD 1.7) hours and men for 11.1 (SD 1.8) hours.

**Table 1:** Sample characteristics of users (n=48,058).

Characteristics	Female	Male	Overall
Sample, n (%)	34,238 (71.24)	13,820 (28.76)	48,058 (100)
Age (y), mean (SD)	49.9 (10)	48.1 (10.2)	50.0 (9.7)
BMI (kg/m^2^), mean (SD)	29.2 (6)	30.4 (5.3)	28.9 (5.6)

### Evaluation Outcomes

**Figure 2:**
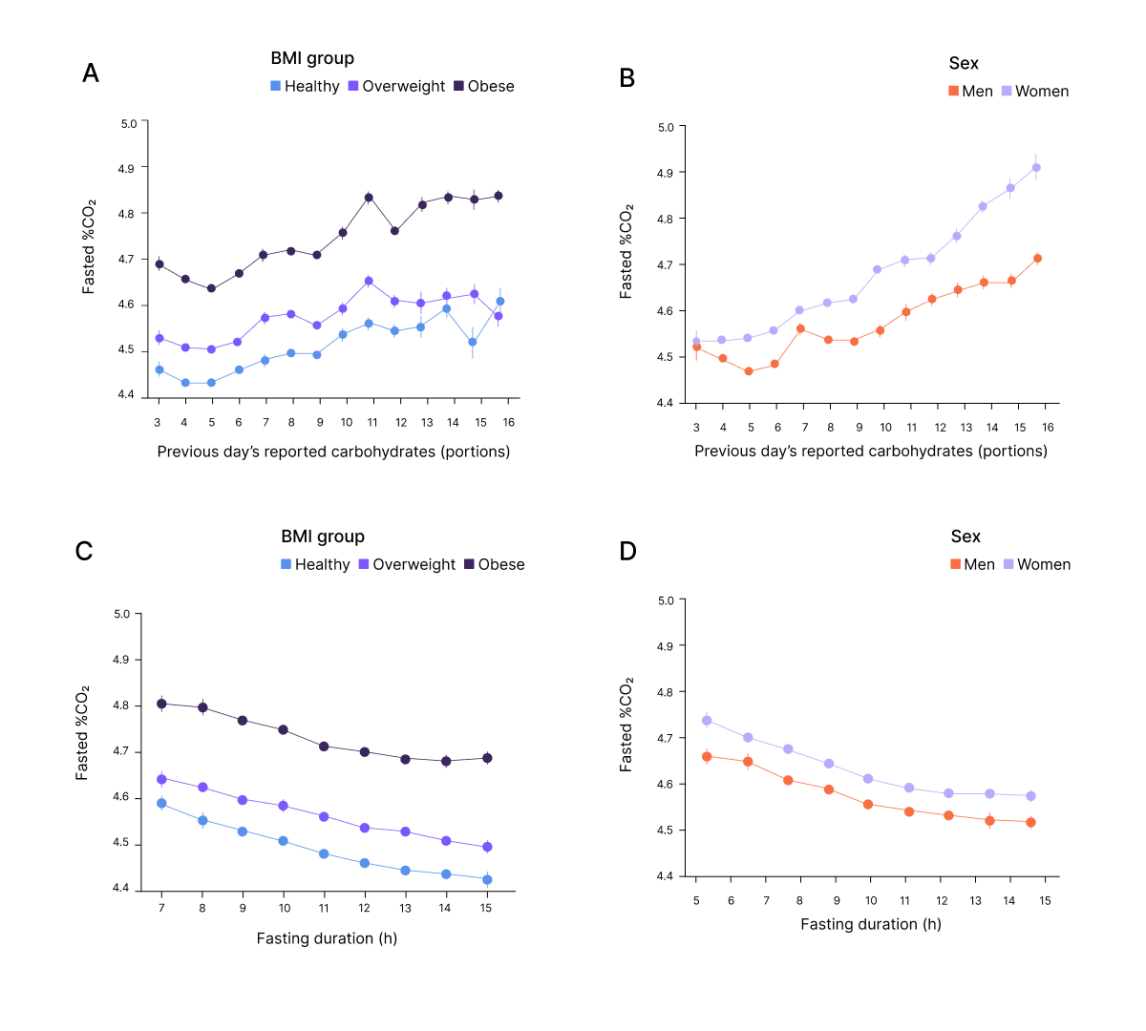
Fasted %CO_2_ measurements in response to different parameters (mean and 95% CI): (A) previous day’s reported carbohydrate consumption, categorized by BMI groups (healthy, overweight, and obese); (B) previous day’s carbohydrate consumption, categorized by sex (male and female); (C) fasting duration, categorized by BMI groups; and (D) fasting duration, categorized by sex.

Figure 2 presents the relationship between fasted %CO_2_ levels and previous day’s reported carbohydrates (A and B) and fasting duration (C and D), categorized by BMI (A and C) and sex (B and D). As expected, increased reported carbohydrate consumption in the previous day resulted in increased %CO_2_, and longer fasting duration resulted in decreased %CO_2_. In addition, users with obesity showed higher %CO_2_ than users with overweight, which in turn had higher %CO_2_ than those with healthy BMI. Moreover, female users displayed higher %CO_2_ than male users, with a mean morning %CO₂ of 4.60% (SD 0.47) in women and 4.56% (SD 0.46%) in men.

The relationship between fasted %CO_2_ levels and previous day’s carbohydrates, fasting duration, and users’ personal information as recorded in the Lumen app was examined using separate linear mixed models for women (Table 2) and men (Table 3). User ID was modeled as a random effect to account for interindividual variability (see full random effects results in Table S1 in Multimedia Appendix 1). In both sexes, higher BMI and reported carbohydrate intake were associated with increased %CO_2_ levels (reported carbohydrate intake: β=.032; *P*<.001; Cohen *d*=0.0691 in women and β=.024; *P*<.001; Cohen *d*=0.0534 in men and BMI: β=.018; *P*<.001; Cohen *d*=0.0390 in women and β=.017; *P*<.001; Cohen *d*=0.0384 in men), while prolonged fasting was linked to decreased %CO_2_ levels (β=−.017; *P*<.001 for both and Cohen *d*=−0.0374 in women and Cohen *d*=−0.0382 in men), corroborating the observations in Figure 2. Cohen *d* values suggest that carbohydrate intake had the strongest effect size, while fasting duration and BMI had relatively smaller effects in both models. Age had a small but statistically significant effect only in women (β=.008; *P*<.001; Cohen *d*=0.0180).

Both models demonstrated good fit, with diagnostic checks supporting their validity. Further details, including Akaike Information Criterion, Bayesian Information Criterion, *R*² values, and full diagnostic results, are provided in Table S1 and Figure S1 in Multimedia Appendix 1.

**Table 2:** Determinants of %CO_2_ in a fasted state in women (n=520,269).

Variables	β (95% CI)	*z* statistics	Cohen *d*	*P* value
Intercept	3.671 (3.642 to 3.700)	253.68	8.0015	<.001
Previous day’s carbohydrates (portions)	0.032 (0.031 to 0.032)	133.00	0.0691	<.001
Fasting duration (h)	−0.017 (−0.0018 to −0.017)	−55.28	−0.0374	<.001
BMI (kg/m^2^)	0.018 (0.017 to 0.019)	53.11	0.0390	<.001
Age (y)	0.008 (0.008 to 0.009)	40.54	0.0180	<.001

**Table 3:** Determinants of %CO_2_ in a fasted state in men (N=187,103).

Variables	β (95% CI)	z statistics	Cohen *d*	*P* value
Intercept	4.010 (3.963 to 4.057)	165.581	8.8970	<.001
Previous day’s carbohydrates (portions)	0.024 (0.023 to 0.025)	85.741	0.0534	<.001
Fasting duration (h)	−0.017 (−0.0018 to −0.016)	−35.173	−0.0382	<.001
BMI (kg/m^2^)	0.017 (0.016 to 0.019)	29.257	0.0384	<.001
Age (y)	0.001 (0.000 to 0.002)	2.317	0.0016	.021

## Discussion

### Principal Findings

This study provides evidence that the Lumen device can detect changes in morning %CO_2_ levels in relation to reported carbohydrate intake from the previous day, with similar metabolic responses observed in male and female Lumen users, apart from a notable age effect in women (Tables 2 and 3). The response is also influenced by fasting duration and BMI to a lesser extent (Figure 2).

Higher reported carbohydrate intake was associated with increased morning %CO_2_ in both women and men (Figure 2) aligning with previous studies showing similar results for RER values measured by metabolic carts [13,16]. In addition, prolonged fasting duration was linked to decreased %CO_2_, highlighting the influence of fasting on metabolic processes, which is consistent with findings on time-restricted feeding [24].

The observed shifts in morning %CO₂ values occurred within a physiologically plausible range; the mean morning %CO₂ was 4.60% (SD 0.47%) in women and 4.56% (SD 0.46%) in men. Although the effect sizes were small, even modest changes on the order of 0.03% to 0.05% may reflect meaningful shifts in metabolic substrate use, particularly in large-scale, real-world datasets, in accordance with our previous retrospective analysis of female users [35]. In addition, in a controlled study, fasted %CO₂ levels increased by approximately 0.11% when comparing high- versus low-carbohydrate diets over a 7-day period, demonstrating that even modest shifts can reflect meaningful metabolic adaptations [27]. These results support the physiological relevance of such variations in exhaled CO₂, particularly in relation to carbohydrate intake and metabolic flexibility.

As shown in our previous research, higher BMI was associated with increased %CO_2_ levels [28], suggesting ameliorated metabolic flexibility in individuals with obesity. While most associations were consistent across both sexes, age demonstrated a stronger effect in female Lumen users compared to men, as older age was correlated with elevated %CO_2_ levels in women. This may be due to differences in menstrual cycle phases and menopause status, as previously observed in Lumen users [35], where eumenorrheic women exhibited lower %CO_2_ levels compared to postmenopausal women, suggesting hormonal influence on exhaled %CO_2_.

### Comparison With Prior Work

The findings of our study align with and expand upon previous research in the field of metabolic health and nutrition. Our observation that higher reported carbohydrate intake is associated with increased morning %CO_2_ is consistent with findings from studies using traditional RER measurements. For instance, Galgani et al [13] demonstrated that carbohydrate overfeeding led to increased RER values, indicating a shift toward carbohydrate oxidation. In addition, this retrospective analysis showed similar results to a previous prospective study using the Lumen device. Roberts et al [27] observed significant changes in exhaled %CO_2_ in response to short-term low and high carbohydrate diets, where high carbohydrate diet resulted in higher fasted %CO_2_ levels. Our study adds to this by demonstrating that these effects are observable in a larger, real-world setting, with variations in carbohydrate intake from the previous day significantly influencing morning %CO_2_ measurements.

The inverse relationship we observed between fasting duration and morning %CO_2_ levels aligns with established literature on the metabolic effects of fasting. Ravussin et al [24] demonstrated that time-restricted feeding increased fat oxidation and decreased RER values in addition to improved metabolic flexibility in overweight adults. Similarly, Vinales et al [15] showed that fasting led to decreased RER values from the metabolic chamber, while high carbohydrate intake resulted in increased RER. As longer overnight fasting was previously associated with reduced BMI [38], this study suggests that Lumen could be a valuable tool for monitoring the metabolic effects of various fasting regimens which might assist in weight loss.

Our study identified significant associations between %CO_2_ levels and various demographic factors, including BMI, age, and sex. The positive correlation between BMI and %CO_2_ levels is consistent with previous research on metabolic flexibility. For instance, Begaye et al [14] found that individuals with higher BMI exhibited impaired metabolic flexibility, which could explain the higher %CO_2_ levels observed in our study. In addition, our previous retrospective analysis demonstrated the relationship between low metabolic flexibility in users with obesity [28].

The observed associations between carbohydrate intake, fasting duration, and exhaled CO₂ may be explained by the role of insulin in substrate oxidation, as carbohydrate intake stimulates insulin secretion, promoting carbohydrate use while suppressing fat oxidation [13,19]. Similarly, the reduction in %CO₂ with prolonged fasting reflects a metabolic shift from carbohydrate to fat oxidation, indicative of improved metabolic flexibility [14,16]. Hormonal responses to fasting, including increased glucagon and cortisol and reduced insulin, further modulate substrate use [24,39]. In women, progesterone fluctuations during the menstrual cycle may also contribute to metabolic flexibility, as higher progesterone levels have been linked to increased lipid oxidation and lower RER values, which could partly explain the observed sex differences in age-related %CO₂ patterns [35,40,41]. Future research should further investigate these hormonal influences to better understand individual metabolic responses.

The observed sex-based differences in our study are particularly interesting in light of our recent work by Cramer et al [35], which explored the relationship between the menstrual cycle and menopause to exhaled CO_2_ in Lumen users. This study showed cyclical %CO_2_ patterns in eumenorrheic women, with lower values compared to menopausal women, suggesting a hormonal influence on metabolic fuel use. Consistently, our findings indicate that age was significantly associated with %CO₂ in women but had a negligible effect in men. Given that most Lumen users analyzed in this study are women, this could explain the stronger association of age with fasted %CO₂ levels in the female cohort. Future research should aim to elucidate the underlying hormonal and physiological mechanisms driving these sex-specific responses and their potential implications for personalized nutrition and health strategies.

Longitudinal investigations into the relationship between exhaled CO_2_ levels and weight loss outcomes could provide crucial insights into the potential of Lumen device for weight management. These studies could explore whether fluctuations in morning %CO_2_ levels serve as correlates of long-term weight loss success. In addition, the applicability of the device should be assessed in diverse populations, including individuals with metabolic disorders such as type 2 diabetes or polycystic ovary syndrome, as well as athletes and older adults, to determine whether metabolic responses differ across various health and lifestyle conditions. Future research should also investigate the integration of Lumen with other health monitoring technologies, such as CGMs or wearable activity trackers, to provide a more comprehensive view of metabolic health. Furthermore, future research should delve into the impact of additional parameters on exhaled CO_2_ levels. For instance, examining both the acute and chronic effects of physical activity on %CO_2_ measurements could yield valuable data as it was previously shown to influence RER levels [42]. Given the established links between sleep and metabolic health [43], investigating how sleep duration and quality influence %CO_2_ levels would also be beneficial. Similarly, exploring the role of stress levels is important, as cortisol is known to affect metabolic processes and was found to be elevated during prolonged fasting [39]. Ultimately, this research could pave the way for more personalized and effective strategies in metabolic health management.

Our study contributes significantly to the growing field of mobile health (mHealth) and ubiquitous health technologies. The use of the Lumen device as a portable metabolic measurement tool aligns with the broader trend of leveraging consumer-grade devices for health monitoring and management. Similar to how CGMs have revolutionized diabetes management [29], our findings suggest that breath-based metabolic monitoring could play a crucial role in personalized nutrition and weight management strategies. The large-scale, real-world nature of our dataset is particularly valuable in the context of mHealth research, as it provides insights into how these technologies perform in everyday settings, outside of controlled laboratory environments. This approach is consistent with recent mHealth studies that have demonstrated the effectiveness of mobile apps in promoting health and managing diseases [30,31]. Furthermore, our study exemplifies the potential of mHealth devices to generate large datasets that can be used to identify patterns and personalize interventions, a key advantage noted in reviews of digital health interventions for weight loss [32]. By demonstrating the ability of Lumen device to detect metabolic changes in response to dietary habits and individual characteristics, our research contributes to the broader goal of empowering users to take greater responsibility for their health through accessible, user-friendly technologies [33].

### Limitations

Despite the large dataset of real-world evidence that was used in this analysis, several limitations need to be mentioned. First, as this is a retrospective and observational study, causal relationships between variables cannot be definitively established. While the results provide valuable insights into associations between carbohydrate intake, fasting duration, and metabolic responses, future research using prospective or experimental designs is needed to strengthen causal inferences. Second, as this analysis is based on real-world data, the reported carbohydrate and the reported fasting duration may be miscalculated or incomplete (recall bias). Future studies should consider integrating objective dietary tracking technologies, such as food-tracking tools, to cross-validate self-reported data and reduce potential recall bias. Moreover, as only reported carbohydrate intake was considered in this analysis, it is difficult to determine if other factors in the meal might influence the %CO_2_ as well, such as fats and protein as well as the type of carbohydrate and the fiber composition. In addition, the macronutrient intake of Lumen users might not be representative of most typical diets, as the Lumen app guides them toward a specific diet, primarily low in carbohydrate, and with most of them on a weight loss journey. As this study has specific user characteristics, caution should be exercised in interpreting these conclusions for a broader audience. Future research should aim to validate findings in more diverse populations, including those with different dietary habits and health goals. Our model showed adequate but imperfect homoscedasticity (Figure S1 in Multimedia Appendix 1). Future studies could use generalized additive or robust regression techniques to address this limitation. Finally, fasted %CO_2_ levels might be affected by other confounding variables, including preexisting conditions, medication use, dietary restrictions, menstrual cycle, and lifestyle factors such as stress, sleep, and physical activity, which were not controlled for in this study. Future studies should control these variables through comprehensive data collection and statistical adjustments, or by designing prospective studies that account for these potential confounders.

### Conclusions

In summary, this study demonstrates that the Lumen device can detect the impact of the previous day’s reported carbohydrate consumption on fasted %CO_2_ measurements in both female and male users. In addition, the device’s sensitivity to variations in fasting duration and BMI suggests its utility in providing personalized dietary recommendations. By accounting for individual differences in metabolic responses, Lumen can offer valuable insights into metabolic health and assist users in optimizing their carbohydrate intake and fasting routines.

## Appendix

Multimedia Appendix 1 Supplementary materials including model fit statistics for the linear mixed models (Table S1) and residual diagnostic plots assessing homoscedasticity, normality, and distribution of residuals (Figure S1).

## Abbreviations

BRANY: Biomedical Research Alliance of New York
CGM: continuous glucose monitoring
IRB: institutional review board
RER: respiratory exchange ratio
