# Supplementary material for: The Association of Previous Day Carbohydrate Consumption With Fasted, Exhaled Carbon Dioxide in Lumen Users: Retrospective Real-World Study
Source: JMIR Mhealth Uhealth. 2025 Sep 2;13:e64604. doi: 10.2196/64604 (PMC12441640; doi:10.2196/64604)
Supplement: Multimedia Appendix 1 [file mhealth_v13i1e64604_app1.doc]

**Supplementary Table S1**

| Model | Autocorrelation (Durbin-Watson statistic) | AIC | BIC | Marginal R² | Conditional R² | Random Effect Variance (User ID) |
| --- | --- | --- | --- | --- | --- | --- |
| Women | 1.99947 | 330,363.3 | 330,441.5 | 0.1120 | 0.6009 | 0.11588 |
| Men | 2.00147 | 116,329.7 | 116,400.7 | 0.0806 | 0.5809 | 0.11055 |

This table reports key model fit statistics for the linear mixed models (LMMs). The Durbin-Watson statistic values near 2 indicate no concerning residual autocorrelation. The Akaike Information Criterion (AIC) and Bayesian Information Criterion (BIC) values indicate model comparison metrics. Marginal R² represents the variance explained by fixed effects, while Conditional R² includes both fixed and random effects. The variance of the random intercept (User ID) captures individual variability in %CO2 measurements.

**Supplementary Figure S1**


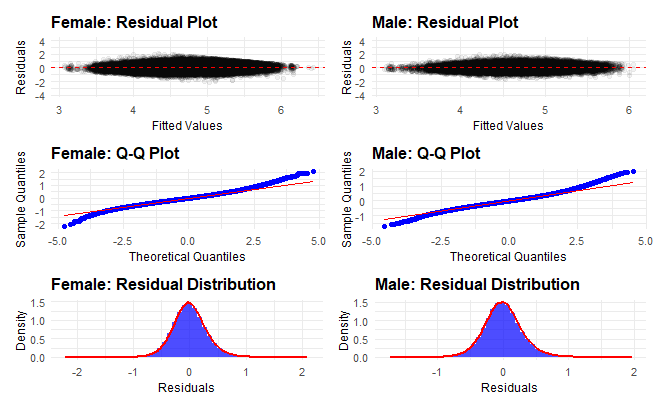


This figure presents residual diagnostic checks for the LMMs to assess model validity:

- Top row: Residual plots for homoscedasticity (ensuring variance is constant across fitted values).
- Middle row: Q-Q plots for normality assessment of residuals.
- Bottom row: Histograms of residual distributions.
